# Simple HPLC-UV Method for Piperacillin/Tazobactam Assay in Human Plasma

**DOI:** 10.3390/antibiotics12020321

**Published:** 2023-02-03

**Authors:** Khaled Abdelkawy, Tyson Le, Sherif Hanafy Mahmoud

**Affiliations:** 1 Faculty of Pharmacy, Kafrelsheikh University, Kafrelsheikh 6860404, Egypt; 2 Faculty of Pharmacy and Pharmaceutical Sciences, University of Alberta, Edmonton, AB T6G 2E1, Canada

**Keywords:** piperacillin, tazobactam, HPLC, plasma, chromatography, assay

## Abstract

Background: Piperacillin (Pip)/tazobactam (Taz) is a broad-spectrum antimicrobial agent that has been commonly used in the intensive care unit for severe and life-threatening infections. Recent evidence suggests that therapeutic drug monitoring (TDM) for Pip could be beneficial in clinical practice to facilitate dose optimization and increase the odds of treatment success. The aim was to develop and validate a sensitive and simple high-performance liquid chromatography (HPLC) method for the simultaneous quantification of Pip and Taz in human plasma. Methods: Samples (0.3 mL) were deproteinized with acetonitrile. The supernatant was evaporated and then reconstituted and injected into the HPLC. The chromatographic analysis was carried out by using the C18 column and gradient elution with the acetonitrile:water mobile phase mixture with 0.1% trifluoracetic acid at a flow rate of 0.8 mL/min using a UV detector at 218 nm. Results: The method had acceptable linearity (r^2^ > 0.99) over the concentration ranges of 0.5–400 μg/mL and 1–100 μg/mL for Pip and Taz, respectively. The method demonstrated acceptable inter- and intra-day precision and accuracy within ±20% with adequate stability results. Conclusion: The developed method is sensitive and simple and utilizes simple sample preparation and elution steps, making it suitable and practical for Pip/Taz TDM.

## 1. Introduction

**Figure 1:**
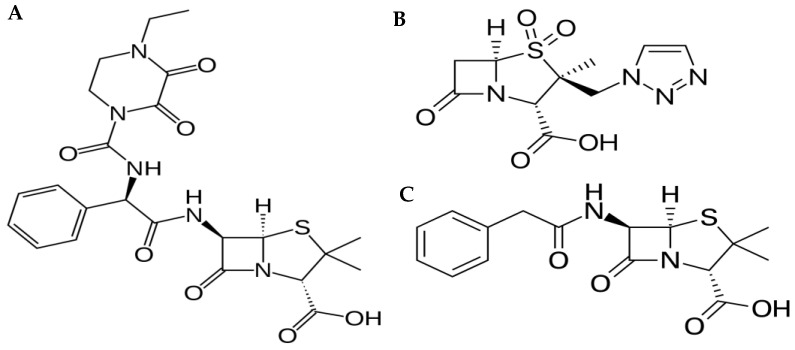
Chemical structures of (**A**) Piperacillin; (**B**) Tazobactam; and (**C**) Penicillin G (internal standard).

Piperacillin/tazobactam is a β lactam/β lactamase inhibitor combination that exhibits a broad spectrum of activity against bacteria (Figure 1). Currently, this combination is widely used in the intensive care unit for managing severe infections such as sepsis [1,2]. Piperacillin (Pip) is a fourth-generation β lactam antimicrobial with antibacterial activity against pathogens such as *Pseudomonas aeruginosa*. The addition of the β lactamase inhibitor, tazobactam (Taz), to Pip greatly enhances Pip’s activity against β lactamase-producing bacteria [1]. Pip/Taz is marketed as an 8:1 fixed-dose combination of 4.0 g/0.5 g, 3 g/0.375 g and 2 g/0.25 g of Pip and Taz, respectively. Pip/Taz bacterial killing is time-dependent, meaning that the bactericidal effect is related to the duration that the antimicrobial concentration is above the minimum inhibitory concentration (MIC) of the pathogen (T > MIC) [3]. Clinically, the therapeutic drug monitoring (TDM) of Pip/Taz is not routinely performed; however, it has been reported that Pip/Taz systemic exposure is highly variable in critically ill patients [4]. To illustrate, critically ill patients with augmented renal clearance exhibit sub-therapeutic concentrations of Pip, potentially resulting in treatment failure and drug resistance [5,6,7,8], suggesting the need for Pip TDM and the need for a simple analytical method for determining Pip/Taz concentration in human plasma [9,10].

Previous analytical methods have been reported to determine Pip and Taz in human plasma samples. These techniques utilized either reverse-phase high-performance liquid chromatography (HPLC) coupled with UV detection [11,12,13,14] or liquid chromatography mass spectrometry (LC–MS) [15,16,17,18]. Although LC-MS is more sensitive than HPLC-UV, MS detectors are not readily accessible in clinical settings due to both economic and technical issues. Additionally, Paal et al. reported that both HPLC-UV and LC-MS methods had comparable results, and HPLC-UV analysis is adequate for Pip TDM [19].

Herein, this study reports a simple and sensitive HPLC-UV method to quantify Pip and Taz in human plasma simultaneously. This method utilizes a simple mobile phase mixture of acetonitrile and water, making it less labor intensive as opposed to previously reported methods that use buffer systems [12,13,14,20]. In addition, sample preparation involves a single step with protein precipitation, simplifying the sample preparation steps of the previously reported methods [20,21]. The method is linear over the Pip concentration range of 0.5–400 μg/mL, covering the concentration ranges reported in critically ill patients, making it suitable and practical for TDM. 

## 2. Results

### 2.1. Method Development

**Figure 2:**
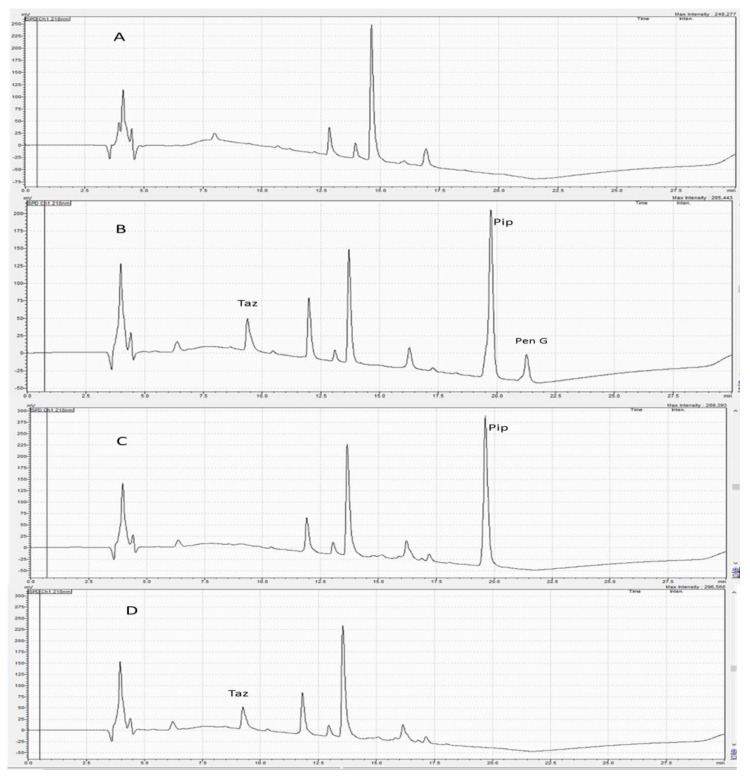
(**A**) A chromatogram of blank plasma; (**B**) a chromatogram of plasma containing piperacillin, tazobactam and penicillin G (IS); (**C**) a chromatogram of plasma sample containing piperacillin only; (**D**) a chromatogram of plasma containing tazobactam only. Retention times determined for tazobactam, piperacillin, and penicillin G (IS) were 9.2, 19.6 and 21.4 min, respectively.

Preliminary experiments were carried out to improve the chromatographic conditions for quantifying Pip and Taz. Pip, Taz, and the internal standard Pen G are hydrophilic compounds with the following order of polarity: Taz, Pip, then Pen G. Therefore, they were expected to elute easily using polar solvents such as acetonitrile and water. Varying mobile phase mixtures of acetonitrile and water at different percentages were tested using isocratic elution. The intention for varying polarities using different mixtures was to separate the three analytes. This method tested the following compositions: 25:75, 50:50, 60:40, 70:30 and 75:25 *v*/*v* acetonitrile:water with different injection volumes and at different flow rates. These combinations did not produce adequate separation of peaks. Specifically, these isocratic trials led to broad, tailing and overlapping peaks for Pip. As a result, the gradient elution of mobile phases containing organic solvents, water and TFA as a modifier with different percentages was carried out. TFA acts to prevent peak tailing and improve peak shape [22]. The best chromatographic conditions were obtained using 0.1% TFA in water and acetonitrile mixtures (as described in the methods section above) at a flow rate of 0.8 mL/min for 22.5 min on a C18 column, using a UV detector at 218 nm. Under these conditions, Pip, Taz, and Pen G (IS) were adequately separated with retention times of 9.2, 19.6, and 21.4 min, respectively (Figure 2). Furthermore, this method tried a few solvents for sample extraction, such as methanol, acetonitrile and hexane. Acetonitrile alone provided superior extraction recovery.

### 2.2. Method Validation

#### 2.2.1. Linearity

**Figure 3:**
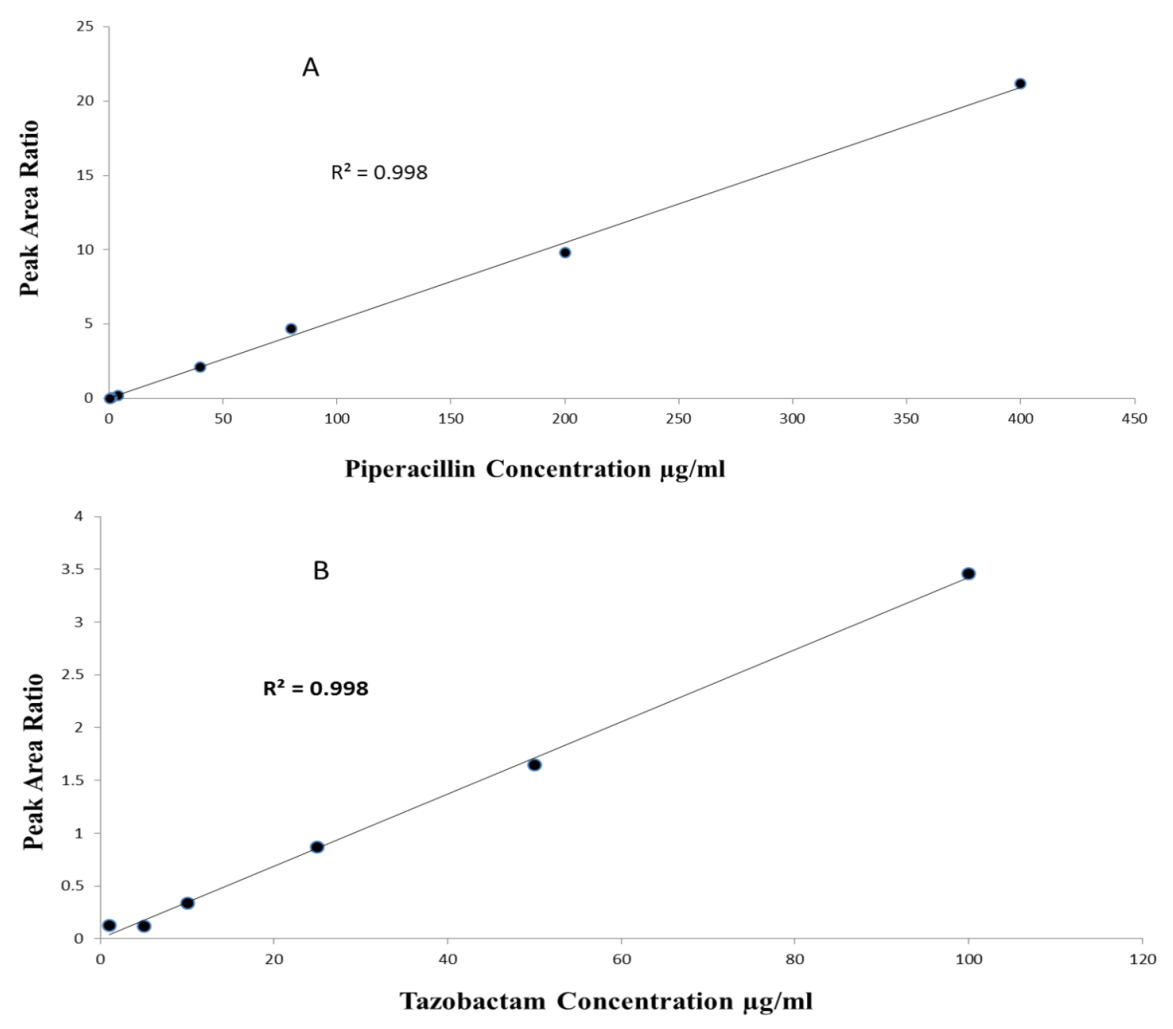
(**A**) Linearity of piperacillin over range of 0.5–400 µg/mL; (**B**) linearity of tazobactam over range of 1–100 µg/mL.

The method linearity was assessed by eluting pure and extracted samples over the tested calibration concentrations. For Pip, the linearity range was 0.5–400 µg/mL with r^2^ > 0.99 (n = 5, *p*-value < 0.0001), with a slope of 0.05 (95%CI 0.051–0.052, *p*-value < 0.0001) and intercept of 0.009 (95%CI −0.07–0.09, *p*-value = 0.781). For Taz, the linearity range was 1–100 µg/mL with r^2^ > 0.99 (n = 5, *p*-value < 0.0001), a slope of 0.03 (95%CI 0.029–0.033, *p*-value < 0.0001) and intercept of 0.07 (95%CI −0.01–0.17, *p*-value = 0.08) (Figure 3).

#### 2.2.2. Selectivity and Sensitivity

As depicted in Figure 2, no interfering peaks were found at both Pip and Taz retention times. Furthermore, no peaks were found at the retention time of the internal standard (Pen G). LLOQ was found to be 0.5 µg/mL for Pip and 1 µg/mL for Taz.

#### 2.2.3. Accuracy and Precision

The intra- and inter-day accuracy and precision of the assay are summarized in Table 1. The intra-day and inter-day precision for both Pip and Taz were less than 20%. The intra-day and inter-day error percentages were within ±20%.

#### 2.2.4. Recovery

We analyzed three QC concentrations to determine extraction recoveries (8, 160, 320 µg/mL for Pip and 20, 40, and 75 µg/mL for Taz). As shown in Table 2, the mean recovery of Pip ranged from 86.13 ± 10% to 90.64 ± 8.96% with a CV of <18%, and Taz’s average recovery ranged from 80 ± 4.25% to 88.84 ± 6.4% with a CV of <18% and the Pen G average recovery was 90.57 ± 11.74% with CV < 13%.

#### 2.2.5. Stability

To determine the stability of Pip, Taz, and Pen G solutions, we determined the accuracy of the predicted concentrations obtained following various storage conditions (Table 3). All drugs were stable during the freeze–thaw cycles and following short-term storage at room temperature for 4 and 12 h. The percent of Pip obtained following these storage conditions ranged from 88.6 to 98.7%. For Taz, the percent of drugs obtained ranged from 88.1 to 98.5%. On the other hand, Pen G exhibited a significant decrease in stability (>20%) at the third freeze–thaw cycle. Additionally, all drugs (Pip, Taz, and Pen G) experienced significant degradation after one week of storage at room temperature (>20%). In contrast, all drugs were stable after one week at 4 °C and −80 °C.

## 3. Discussion

This study describes a reliable, simple and sensitive method for measuring Pip and Taz in human plasma. This method has many advantages compared to the previously reported methods for Pip assay. First, the method has high sensitivity, as indicated by an LLOQ of 0.5 µg/mL with a wide linearity range over the concentrations reported for Pip in critically ill patients [23]. Second, one extraction step with adequate extraction recovery is used for sample preparation. Third, no phosphate or acetate buffer was used in the mobile phase, eliminating the risk of precipitation within the HPLC system. Although Pip/Taz TDM is merely related to the Pip concentrations, this method elected to simultaneously determine Taz concentration to confirm no interference during chromatographic elution, making this method practical for future research. 

In this study, Pen G was chosen as an internal standard as it has similar physicochemical characteristics compared to Pip and Taz. Pip, Taz, and Pen G are hydrophilic with the following order of polarity: Taz, Pip, then Pen G. All the drugs are soluble in water, ethanol, and methanol. Pen G eluted after both Taz and Pip because it is less polar than the analytes [24]. Although we implemented a relatively long run time (22.5 min) using a 0.8 mL/min flow rate, this flow rate with the gradient elution conditions enabled the clear separation of peaks without any peak overlapping as opposed to a higher flow rate with short run-time conditions. In addition, this method uses a relatively small injection volume for HPLC analysis assay (20 µL) as opposed to the 50–75 µL injection volume utilized in previous methods [11,21]. 

Most mobile phase compositions for the previously reported methods used phosphate or acetate buffer systems [12,13,14,20]. The main issue with using a phosphate or acetate buffer in the mobile phase is that they can precipitate in the HPLC pump, which requires a lot of work to clean out, and parts may even have to be replaced. Second, the buffer may precipitate inside the column, necessitating column replacement, because buffer precipitates in the pores of the stationary phase. This precipitation is more pronounced with acetonitrile used as the mobile phase [25]. In this method, adding TFA to the mobile phase kept the pH below the pKa of both Pip (pKa 3.4) and Taz (pKa 2.86), resulting in sharper peaks and eliminating the precipitation risk associated with phosphate or acetate buffers [26].

One key advantage of this method is the wide linearity range (0.5–400 µg/mL) which covers the reported Pip concentration in clinical practice [23,27]. Calibration concentrations were selected to cover the reported concentration ranges in clinical practice. Since Pip/Taz is dosed in an 8:1 ratio (e.g., 4 g Pip and 0.5 g Taz), Pip has higher reported concentrations (that could exceed 100 µg/mL) than tazobactam (7–40 µg/mL) [28,29]. Therefore, a wider calibration curve for the Pip was selected. Previous methods had an upper limit of quantification for Pip that is <100 µg/mL, which might not cover concentrations in patients where Pip concentrations >100 µg/L have been reported [15,16,30,31,32]. This advantage gives priority to the routine use of this method in clinical situations such as kidney dysfunctions, where patients are expected to show a higher concentration than normal populations. On the other hand, other methods had lower sensitivity than this method, precluding the detection of Pip in patients with low Pip concentrations, such as those with augmented renal clearance [33,34,35]. 

For routine analysis in clinical practice, sample preparation should be easy and cost-effective. The method provides a one-step acetonitrile addition for sample preparation with good recovery, simplifying this analysis method.

The stability of Pip is a very important part of validation as β lactam antibiotics are quite unstable if kept at room temperature for a long time [36]. Validation should ensure that stability limitation should not affect Pip monitoring in clinical practice, and strict recommendations concerning sample transmission and correct storage conditions should be warranted. The stability results showed that both Pip and Taz have sufficient stability for laboratory analysis if stored in a refrigerator or at −80 °C or kept at room temperature for 12 h. These stability results are in agreement with previous stability results of Pip methods [11,21], where Pip was stable at room temperature for 12 h and after freeze–thaw cycles.

## 4. Materials and Methods

### 4.1. Reagents and Chemicals

Standard Pip, Taz, and the internal standard (IS) Penicillin G (Pen G) were purchased from Sigma-Aldrich (Oakville, ON, Canada). Figure 1 shows the chemical structures of these compounds. HPLC-grade acetonitrile, water and methanol were obtained from Fisher Scientific (Edmonton, AB, Canada). Trifluoracetic acid (TFA) analytical grade was purchased from Sigma-Aldrich (Oakville, ON, Canada), and pooled human plasma was obtained from Cedarlane Laboratories (Burlington, ON, Canada).

### 4.2. Instrument

The experiment was carried out using the Shimadzu LC 20A HPLC system (Shimadzu, Kyoto, Japan). The system is comprised of a system controller (CBM-20A), an HPLC binary pump (LC-10 AD), an autosampler (SIL-10-AF), and a UV-VIS detector (SPD-10A). Elution was performed by using a C18 reverse phase Supleco Discovery^®^ C18 column (5 μm, 250 × 4.6 mm) (Supleco Inc, Mississauga, ON, Canada) with a Discovery^®^ C18 Supelguard™ guard column (5 μm, 20 × 4 mm) (Supleco Inc., Mississauga, ON, Canada). LabSolutions^®^ software (Shimadzu, Kyoto, Japan) was used for data collection and chromatographic analysis.

### 4.3. Chromatographic Conditions

Analytes were eluted using a gradient elution of a mixture of mobile phase A: consisting of 90% HPLC-grade acetonitrile and 10% HPLC-grade water with 0.1% TFA and mobile phase B: 97% HPLC-grade water with 0.1% TFA and 3% HPLC-grade acetonitrile. The gradient elution conditions are shown in Table 4 using a flow rate of 0.8 mL/min for 22.5 min. Analytes were detected at a wavelength of 218 nm.

### 4.4. Pip and Taz Stock and Working Solutions

In total, 9.6 mg of Pip was weighed using an analytical balance (Mettler Toledo XSR64, Greifensee, Switzerland) and dissolved in 3 mL of 60:40 *v*/*v* HPLC-grade water: methanol to prepare a solution of 3.2 mg/mL. Similarly, 2 mg of Taz was weighted and dissolved in 5 mL of 60:40 (*v*/*v*) HPLC grade water: methanol to obtain a solution of 400 µg/mL using a Vortex mixer (Fisher Vortex Mixer Genie 2, Fisher Scientific, Bohemia, NY, USA). Working solutions were prepared by mixing equal volumes of the prepared Pip and Taz stock solutions together to obtain a working solution containing 1600 µg/mL Pip and 200 µg/mL Taz (8:1). A working IS solution was obtained by dissolving Pen G powder in HPLC-grade water to obtain 1050 µg/mL. All solutions were freshly prepared daily.

### 4.5. Pip and Taz Calibration Concentrations and Quality Control (QC) Samples

Calibration standards for Pip were prepared in blank human plasma at different serial dilutions of the standard working solutions to prepare final concentrations of 0, 0.25, 0.5, 1, 4, 40, 80, 200, and 400 µg/mL of Pip. Four quality control (QC) samples were obtained by spiking blank plasma with Pip for method validation. The prepared QC samples contained the lowest limit of quantification (LLOQ), a low-level QC containing 8 µg/mL (QC1), a middle-level QC containing 160 µg/mL (QC2), and a high-level QC containing 320 µg/mL (QC3) of Pip. 

Similarly, calibration standards for Taz were prepared in blank human plasma at different dilutions of the standard working solutions to obtain final concentrations of 0, 0.5, 1, 5, 10, 25, 50, and 100 µg/mL of Taz. Four quality control (QC) samples were prepared by spiking drug-free human plasma with Taz for method validation. The prepared QC samples contained the lowest limit of quantification (LLOQ), a low-level QC containing 20 µg/mL (QC1), a middle-level QC containing 40 µg/mL (QC2), and a high-level QC containing 75 µg/mL (QC3) of Taz. 

### 4.6. Sample Preparation

In total, 300 µL of human plasma spiked with Pip and Taz was added to 50 µL of 1050 µg/mL Pen G (IS). Samples were then vortex mixed for 45 s to ensure homogenous mixing. Then, 1.5 mL acetonitrile was added to the samples, followed by another vortex mixing procedure for 1 min. Samples were centrifuged at 5000 rpm for 10 min (Eppendorf centrifuge 5804, Eppendorf SE, Barkhausenweg, Hamburg, Germany). Then, the supernatant was transferred to clean tubes and evaporated by a SpeedVac^®^ Vacuum Concentrator (Thermo Fisher Scientific, Waltham, MA, USA). The remaining residue was reconstituted with 300 µL of mobile phase mixture (5% of mobile phase A and 95% of mobile phase B), and 20 µL of the reconstituted sample was injected into the HPLC.

### 4.7. Method Validation

Method validation procedures were performed according to the FDA Bioanalytical Method Validation Guidance [37]. Method validation involved linearity, selectivity, sensitivity, precision, accuracy, stability, and extraction recovery.

#### 4.7.1. Linearity

The linearity of the method was determined by creating calibration curves by plotting peak area ratios (Pip/Pen G as IS) vs. the calibration concentrations. Likewise, Taz linearity was assessed by constructing calibration curves by plotting peak area ratios (Taz/Pen G as IS) vs. the calibration concentrations. Linear regression was performed to calculate the coefficient of determination (r2), the slope, and the intercept of the regression line. Statistics (F-test for regression and t-statistic for the slope and the intercept) were conducted using STATA version 15.1 (StatCorp, College Station, TX, USA). 

#### 4.7.2. Selectivity and Sensitivity

Blank plasma samples were injected prior to each run. We assessed the method’s selectivity according to the absence of peaks at the retention times of Pip, Taz, and IS when blank plasma samples were eluted. LLOQ had the lowest concentration, with a calculated precision of < 20% and accuracy of within ±20%. Furthermore, the signal of the LLOQ peak should be 5 or more times higher than the blank plasma signal. 

#### 4.7.3. Accuracy and Precision

The intra- and inter-day accuracy and precision of the assay were determined by assaying 5 replicates of QC samples spiked with Pip at the 3 QC levels (8, 160, 320 µg/mL) in addition to LLOQ on three different days. In a similar way, Taz’s accuracy and precision were attained by assaying five replicates of QC samples spiked with the 3 QC levels for Taz (20, 40, and 75 µg/mL, respectively) in addition to LLOQ on 3 different days. Precision is estimated as the percent coefficient of variation (CV %), and the accuracy is estimated as percent error (%, error).

#### 4.7.4. Recovery

The Pip and Taz extraction recoveries were obtained by dividing the peak areas of the extracted samples of 3 QC samples with those obtained from extracted blank plasma containing equal concentrations of Pip and Taz post extraction.

#### 4.7.5. Stability

Stability studies were conducted to determine the stability of Pip and Taz in human plasma under different storage conditions. For Pip, the experiments were carried out at two concentrations (160 and 320 µg/mL, n = 5) and for Taz, two concentrations were utilized (20 and 40 µg/mL, n = 5). For short-term stability, samples were stored at room temperature (RT) for 4 and 12 h. Three freeze–thaw cycles were also performed, where samples were stored at −20 °C before thawing at RT. The long-term stability was studied at room temperature, 4 °C and −80 °C for one week. The mean peak areas of the analytes were compared to those of freshly prepared samples with equivalent concentrations of the analytes (n = 5), and the percentage of the drug that remained was determined. Samples were considered stable if the accuracy was within 80-120% of the normal values.

## 5. Conclusions

A fully validated HPLC-UV method for determining Pip and Taz in human plasma was presented. The method provides an acceptable linearity range, precision and accurate results with adequate stability. The developed method is simple, sensitive and utilizes simple sample preparation and elution steps, making it suitable and practical for Pip/Taz TDM.

## Figures and Tables

**Table 1:** Intra-day (n = 5) and inter-day precision and accuracy data for the quantitation of piperacillin and tazobactam in human plasma.

	Piperacillin							Tazobactam					
Nominal Conc.	Intra-Day (n = 5)			Inter-Day (n = 15)			Nominal Conc.	Intra-Day (n = 5)			Inter-Day (n = 15)		
(µg/mL)	Conc.* Mean ± SD	CV (%)	% Error	Conc. Mean ± SD	CV (%)	% Error	(µg/mL)	Conc. Mean ± SD	CV (%)	% Error	Conc. Mean ± SD	CV (%)	% Error
0.5	0.51 ± 0.08	15.63	−2.10	0.54 ± 0.03	4.97	−7.95	1	1.18 ± 0.11	9.56	−18.36	1.16 ± 0.02	1.84	−16.21
8	7.68 ± 0.54	7.00	4.00	7.2 ± 0.51	7.11	10.29	20	19.30 ± 3.46	17.92	3.50	21.93 ± 3.31	15.08	−9.63
160	166.73 ± 25.03	15.01	−4.20	172.64 ± 15.76	9.13	−7.90	40	35.45 ± 3.04	8.58	11.37	42.99 ± 6.89	16.01	−7.48
320	286.00 ± 50.55	17.67	10.62	338.53 ± 45.68	13.49	−5.79	75	78.53 ± 6.53	8.32	−4.71	73.36 ± 4.48	6.11	2.19

*: Concentration, CV: Coefficient of variance, SD: Standard variation.

**Table 2:** Extraction recovery for piperacillin, tazobactam and penicillin G.

Recovery Percent (n = 5)								
Nominal Conc. *	Piperacillin % Recovery		Nominal Conc.	Tazobactam % Recovery		Nominal Conc.	Penicillin G % Recovery	
(µg/mL)	Mean ± SD	CV (%)	(µg/mL)	Mean ± SD	CV (%)	(µg/mL)	Mean ± SD	CV (%)
8	90.64 ± 8.96	9.88	20	84.61 ± 14.41	17.0	150	90.57 ± 11.74	12.97
160	87.86 ± 15.16	17.25	40	80.00 ± 4.25	5.34			
320	86.13 ± 10.00	11.61	75	88.84 ± 6.40	7.21			

*: Concentration, CV: Coefficient of variance, SD: Standard variation.

**Table 3:** Stability of piperacillin, tazobactam and penicillin G.

% Remaining		Freeze–Thaw Cycles			Short term Stability (Room Temperature)		Long Term Stability		
							Room Temperature	Refrigerator at 4 °C	Freeze at −80 °C
		Cycle 1	Cycle 2	Cycle 3	4 h	12 h	Week 1	Week 1	Week 1
QC1	Piperacillin	96	92.9	90.1	97.5	95.7	68.4	94.3	95.9
	Tazobactam	95.7	90.1	88.1	96.4	92.8	73.1	96.6	94.9
	Penicillin G	95.7	86.9	79	97.6	92.1	54.4	93.5	96
QC2	Piperacillin	97.6	90.2	88.6	98.7	96.4	71.2	93.5	97
	Tazobactam	98.4	98.5	91.4	94.6	93.4	70.5	97.2	96.4
	Penicillin G	87.2	82.1	76	98.7	93.8	62.8	87.6	95.5

QC: Quality control, h: hour, % remaining: percent drug remaining.

**Table 4:** Gradient elution mobile phase conditions.

Running Time (min)	Mobile Phase B Percentage	Mobile Phase A Percentage
0.00	5	95
3.75	15	85
7.50	25	75
13.25	35	65
15.00	45	55
18.75	25	75
22.50	5	95

Mobile phase A: 90% acetonitrile and 10% water with 0.1% TFA. Mobile phase B: 97% water with 0.1% TFA and 3% acetonitrile.

## Data Availability

All relevant data are within the manuscript.
